# Immunocompatibility of a new dual modality contrast agent based on radiolabeled iron-oxide nanoparticles

**DOI:** 10.1038/s41598-021-89117-3

**Published:** 2021-05-07

**Authors:** Maria-Argyro Karageorgou, Dimosthenis Stamopoulos

**Affiliations:** 1 Department of Physics, National and Kapodistrian University of Athens, Zografou Panepistimioupolis, 15784 Athens, Greece; 2 Institute of Nuclear and Radiological Sciences and Technology, Energy and Safety, National Center for Scientific Research “Demokritos”, Ag. Paraskevi, 15310 Athens, Greece; 3 Institute of Nanoscience and Nanotechnology, National Center for Scientific Research “Demokritos”, Ag. Paraskevi, 15310 Athens, Greece

**Keywords:** Biophysics, Nanoscience and technology

## Abstract

Radiolabeled magnetic nanoparticles are promising candidates as dual-modality-contrast-agents (DMCA) for diagnostic applications. The immunocompatibility of a new DMCA is a prerequisite for subsequent in vivo applications. Here, a new DMCA, namely Fe_3_O_4_ nanoparticles radiolabeled with ^68^Ga, is subjected to immunocompatibility tests both in vitro and in vivo. The in vitro immunocompatibility of the DMCA relied on incubation with donated human WBCs and PLTs (five healthy individuals). Optical microscopy (OM) and atomic force microscopy (AFM) were employed for the investigation of the morphological characteristics of WBCs and PLTs. A standard hematology analyzer (HA) provided information on complete blood count. The in vivo immunocompatibility of the DMCA was assessed through its biodistribution among the basic organs of the mononuclear phagocyte system in normal and immunodeficient mice (nine in each group). In addition, Magnetic Resonance Imaging (MRI) data were acquired in normal mice (three). The combined OM, AFM and HA in vitro data showed that although the DMCA promoted noticeable activation of WBCs and PLTs, neither degradation nor clustering were observed. The in vivo data showed no difference of the DMCA biodistribution between the normal and immunodeficient mice, while the MRI data prove the efficacy of the particular DMCA when compared to the non-radiolabeled, parent CA. The combined in vitro and in vivo data prove that the particular DMCA is a promising candidate for future in vivo applications.

## Introduction

Superparamagnetic iron oxide nanoparticles (IONPs), namely magnetite (Fe_3_O_4_) and/or maghemite (γ-Fe_2_O_3_), are commonly used in various biomedical applications, such as magnetic resonance imaging,^1,2^ hyperthermia cancer treatment,^3,4^ cell separation^5^ etc. due to their exceptional nanoscale physicochemical properties. Among their numerous applications, the use of IONPs in dual modality imaging has attracted significant attention in the last decade^6,7^. Particularly, dual modality contrast agents (DMCA), such as radiolabeled IONPs, are promising candidates in imaging applications since they combine the advantages of two different imaging modalities, for example of Positron Emission Tomography (PET) and/or Single-Photon Emission Computed Tomography (SPECT) with Magnetic Resonance Imaging (MRI) in a way that the obtained results are superior to the sum of those of the two former imaging techniques.

For diagnostic purposes, intravenous injection of a DMCΑ constitutes the most common route of administration. However, following the intravenous injection into blood circulation, plasma proteins are immediately adsorbed onto the surface of the DMCA, resulting in the recognition and interaction of the DMCA with immune cells, namely white blood cells (WBCs)^8^. Apart from WBCs, the circulating DMCA interacts with other important blood constituents namely the coagulation blood cells, that is platelets (PLTs)^9^, whose major contribution is on hemostasis. Thus, before the use of a new DMCA in diagnostic applications, the investigation of its biocompatibility with the aforementioned human blood cells should be a principal concern, in order to avoid any potential side effect that could be hazardous for in vivo use. For this reason, various immunological studies have been reported in the literature highlighting the impact of nanoparticles with different physico-chemical properties (i.e. size, shape, surface charge etc.) on the WBCs and PLTs^8–16^. Particularly, nanoparticles interfere with the biological functions of WBCs (i.e. phagocytosis, oxidative burst etc.), leading to cytotoxic effects such as cellular membrane damage, degranulation, morphological transformation, or even apoptosis^15–20^. Moreover, nanoparticles can hamper the physiological function of PLTs by altering activation and aggregation processes, resulting in acute thrombosis or other detrimental health effects^8,21–26^.

To that effect, the current experimental study refers to the evaluation of the immunocompatibility of a new DMCA that is based on radiolabeled IONPs. Specifically, Fe_3_O_4_ NPs are surface-functionalized with 2,3-dicarboxypropane-1,1-diphosphonic acid (DPD) and subsequently radiolabeled with Gallium-68 (^68^Ga) so that the ^68^Ga-DPD–Fe_3_O_4_ complex is ultimately formed^27^. The immunocompatibility of this DMCA is evaluated here for the first time, both in vitro and in vivo. Referring to the in vitro evaluation, the immunocompatibility was tested upon incubation with human WBCs and PLTs isolated from donated peripheral blood. Referring to the in vivo evaluation, comparative biodistribution studies were performed in normal and immunodeficient mice models. Additionally, in vivo MRI imaging was performed in normal mice. Previously reported studies^28–31^ on relevant IONPs have recruited both optical microscopy (OM) and atomic force microscopy (AFM), in order to investigate, at the microscopic and at the nanoscopic level respectively, the overall morphological and surface membrane characteristics of isolated red blood cells (RBCs). In the same context^31^, samples of both WBCs and PLTs, isolated from donated peripheral blood of five healthy individuals, were incubated at room temperature at tolerable (C_DMCA_ = 0.1 mg/ml) and extreme (C_DMCA_ = 1 mg/ml) concentrations^26^ of ^68^Ga-DPD-Fe_3_O_4_ DMCA, up to 120 min. OM and AFM were recruited to investigate in vitro the morphological characteristics of WBCs and PLTs at the microscopic (10^–6^ m) and at the nanoscopic (10^–9^ m) level, since morphological transformation in both shape and size is a key factor that indicates the activation process for both blood cells. In addition, a standard hematology analyzer (HA), employed in every day clinical practice, was used to obtain standard information on complete blood count for comparative reasons with the results originating from the microscopy techniques. Referring to the in vivo evaluation, we studied the accumulation of the DMCA on the basic organs of the mononuclear phagocyte system, namely liver, spleen and lungs, by injecting a standard DMCA dose via the tail vein in nine normal and nine immunodeficient mice.

In brief, the combined OM and AFM results proved that the presence of the ^68^Ga-DPD-Fe_3_O_4_ DMCA enhances the activation of WBCs and PLTs, as observed at the microscopic level by morphological transformation referring to both their shape and size. Specifically, both WBCs and PLTs undergo a shape change from a completely spherical to a polarized and/or ruffled form with the occasional appearance of pseudopods in the case of PLTs. In addition, the WBCs exhibit a statistically significant size change. Interestingly, at the nanoscopic level we observed that the size of the WBCs’ granules was increased in the activated state. Although WBCs and PLTs are somehow activated by the DMCA, neither degradation nor clustering were observed. Furthermore, the HA data indicated that despite their obvious activation, all standard WBCs’ and PLTs’ indices remained in the physiological range. The results obtained from the in vivo biodistribution study performed between normal and immunodeficient mice practically showed no difference of the DMCA accumulation among the basic organs of the mononuclear phagocyte system. Thus, we can assume that the particular DMCA could potentially evade the immune system. In vivo MRI imaging data were in accordance with the respective in vivo biodistribution data and revealed that the contrast effect produced by the particular DMCA was sufficient enough compared to the one produced by the non-radiolabeled CA, however being concentration-dependent.

Taking into account both the in vitro and in vivo experimental results we propose that the particular DMCA exhibits noticeable potential for future use in diagnostic applications.

## Results and discussion

**Figure 1:**
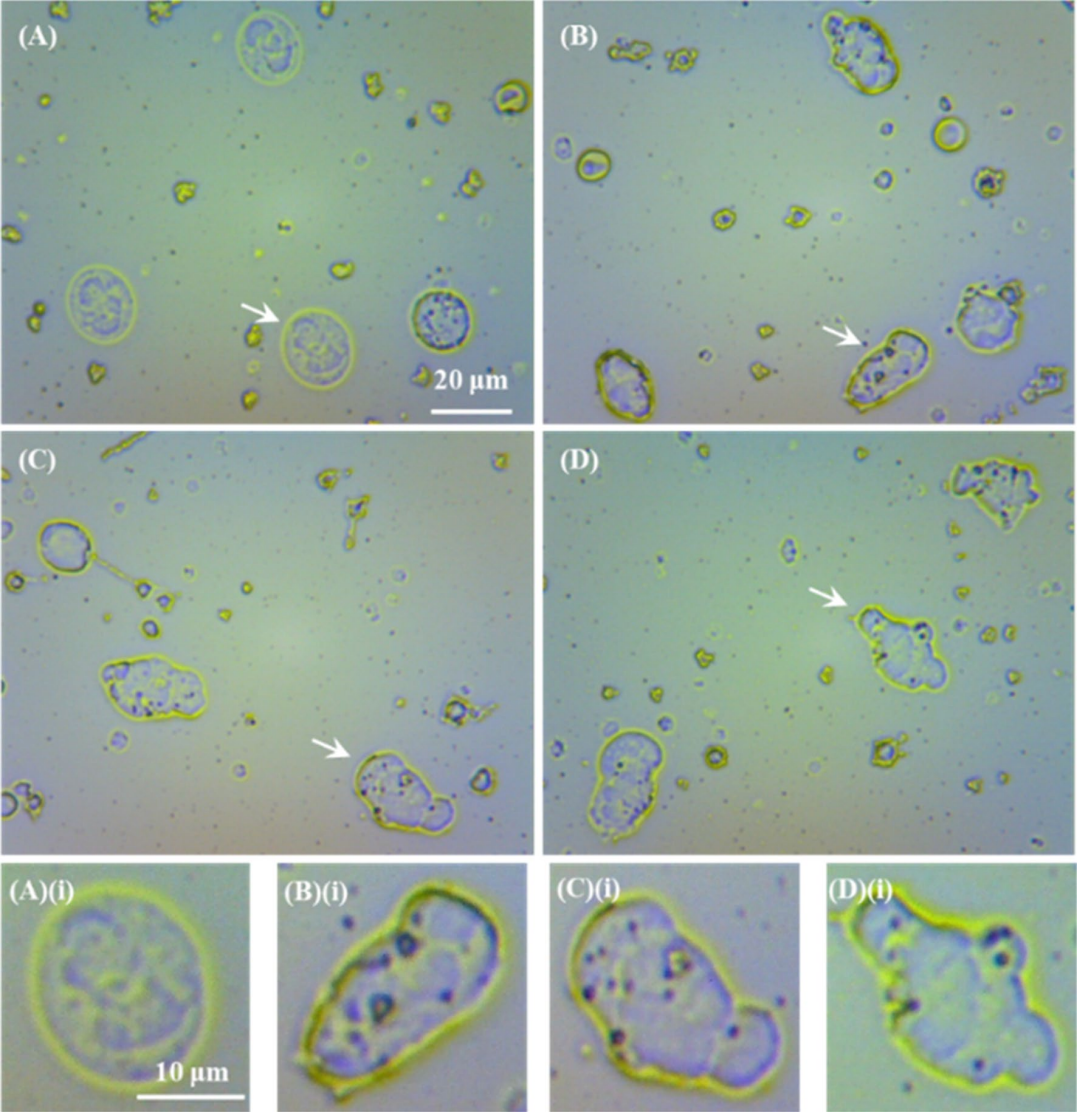
Representative optical microscopy (OM) images of WBCs incubated with and without the DMCA. (**A**–**D**) OM images of WBC films referring to (**A**) DMCA-free prior to incubation (0 min), (**B**) DMCA-free after incubation (120 min), (**C**) DMCA-incubated at C_DMCA_ = 0.1 mg/ml(120 min) and (**D**) DMCA-incubated at C_DMCA_ = 1 mg/ml (120 min) (objective lens × 20, camera × 13 in all cases). (**A**)(**i**)–(**D**)(**i**) Bottom figures show magnification of specific WBCs indicated by the arrows in the respective top figures, in (**A**)(**i**) resting (spherical shape) and in (**B**)(**i**)–(**D**)(**i**) activated state (polarized and/or ruffled shape).

Figure 1A–D show representative OM images of WBC films coming from donor E for DMCA-free prior to (panel (A)) and after 120 min incubation (panel (B)) and DMCA-incubated WBCs for 120 min at room temperature in tolerable and extreme concentrations, C_DMCA_ = 0.1 mg/ml (panel (C)) and C_DMCA_ = 1 mg/ml (panel (D)), respectively. The direct comparison of these images shows that before the incubation process (panel (A)) the WBCs are in resting form, while after the 120 min incubation without (panel (B)) and with the presence of the DMCA (panels (C) and (D)) the WBCs are activated, exhibiting a change of their morphological characteristics, namely shape and size.

In particular, referring to their shape change, panel (A)(i) focuses on a WBC of panel (A) that is in resting state thus preserving its spherical shape, coming from a DMCA-free sample prior to (0 min) incubation. On the contrary, panels (B)(i)–(D)(i) focus on WBCs in activated state of the respective panels (B)–(D), coming from DMCA-free after 120 min incubation (panel (B)(i)) and DMCA-incubated for 120 min samples at C_DMCA_ = 0.1 mg/ml(panel (C)(i)) and at C_DMCA_ = 1 mg/ml (panel (D)(i)) that show the progression of shape transformation from a simple polarized form (panel (B)(i)) to a complex ruffled shape (panel (D)(i)). The same behavior was recorded in all five donors studied here.

**Figure 2:**
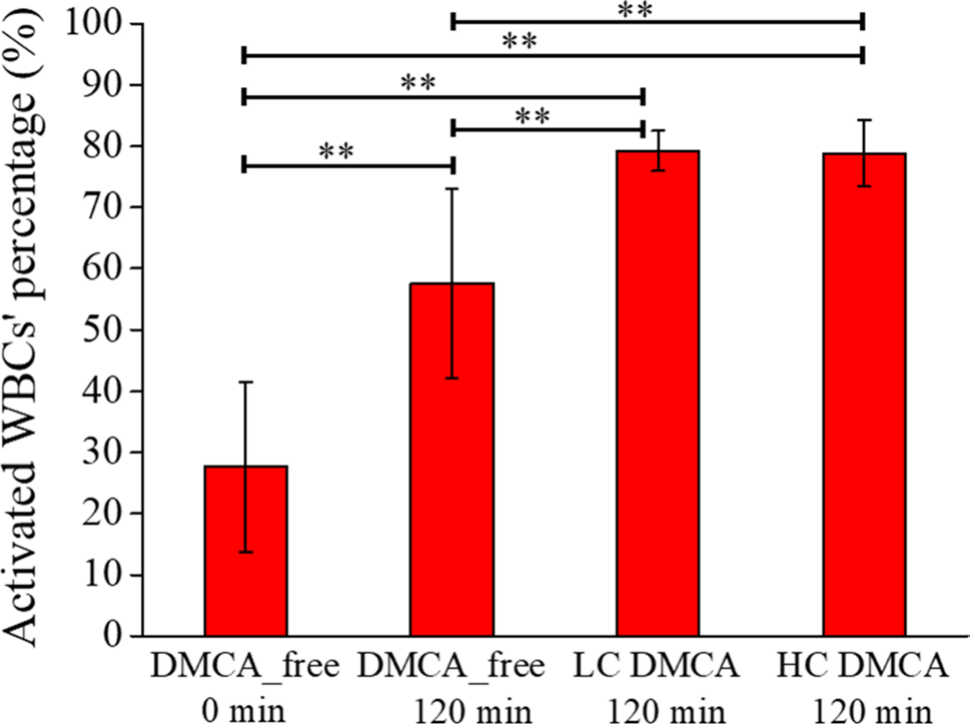
Percentage [%] of activated WBCs’ observed in samples incubated with and without the DMCA. Mean value of the activated WBCs’ percentage [%] observed in DMCA-free (prior to (0 min) and after (120 min) incubation) and DMCA-incubated (C_DMCA_ = 0.1 mg/ml; low concentration—LC and C_DMCA_ = 1 mg/ml; high concentration—HC) samples. In all panels the results are expressed as the overall MV ± SD of the data coming from all five donors.

Figure 2 quantitatively summarizes the OM results referring to the shape change of the activated WBCs observed in the DMCA-free and DMCA-incubated samples with different DMCA concentrations (C_DMCA_ = 0.1 mg/ml; low concentration—LC and C_DMCA_ = 1 mg/ml; high concentration—HC). Specifically, we observed that the WBCs showed a statistically significant activation during 120 min incubation both without DMCA (27.6 ± 13.8% prior to versus 57.6 ± 15.5% after incubation) and with DMCA (79.2 ± 3.3% for C_DMCA_ = 0.1 mg/ml and 78.8 ± 5.4% for C_DMCA_ = 1 mg/ml). Thus, we can assume that at the microscopic level the activation of WBCs is influenced by the incubation process, whereas it is enhanced by the presence of the DMCA, however without being concentration dependent.

**Figure 3:**
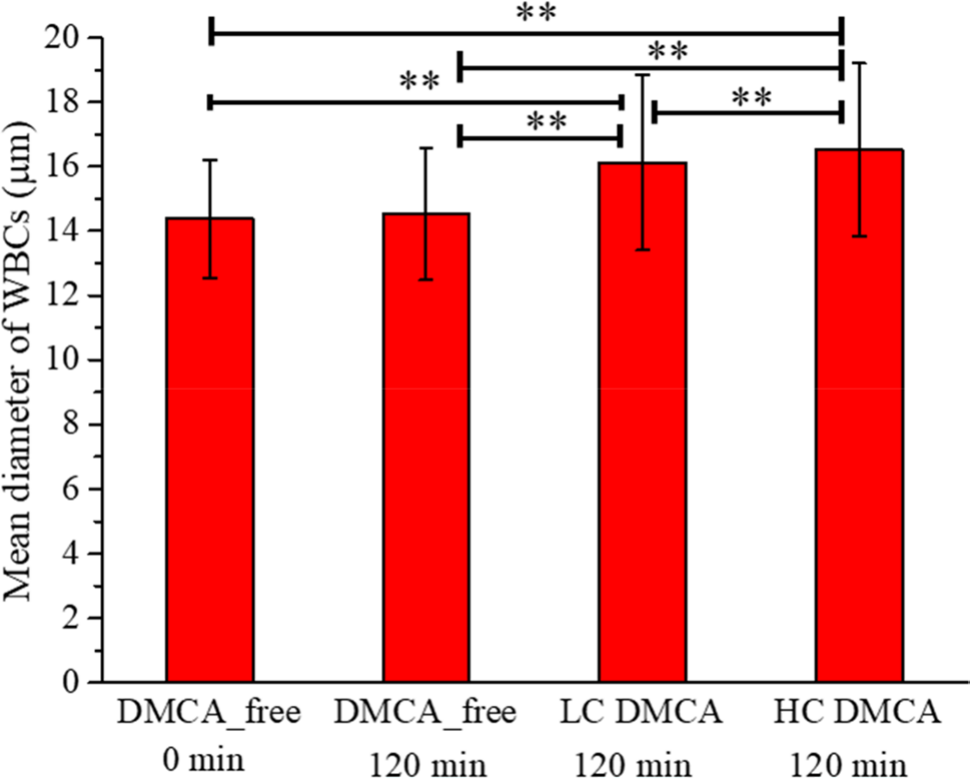
Mean value of the WBCs’ ‘mean diameter’ measured in all five donors by OM. Mean value of the WBCs’ ‘mean diameter’, measured in DMCA-free (prior to (0 min) and after (120 min) incubation) and DMCA-incubated (C_DMCA_ = 0.1 mg/ml; low concentration—LC and C_DMCA_ = 1 mg/ml; high concentration—HC) samples. In both panels the results are expressed as the overall MV ± SD of the data coming from all five donors.

Referring to the size change of WBCs, Fig. 3 quantitatively summarizes the ‘mean diameter’ of the WBCs measured in the DMCA-free and DMCA-incubated samples with different DMCA concentrations (C_DMCA_ = 0.1 mg/ml; low concentration—LC and C_DMCA_ = 1 mg/ml; high concentration—HC). First, we clarify that in activated, elliptical-like WBCs the parameter ‘mean diameter’ refers to the mean value of the two diameters as measured along the basic axis of elongation and the normal direction. Thus, in non-activated, spherical WBCs the ‘mean diameter’ coincides to the typical diameter (for details, see the “Definition of activated cells” in “Methods”). We observed that the ‘mean diameter’ of the DMCA-free WBCs remains practically identical upon incubation (14.38 ± 1.84 μm prior to incubation and 14.53 ± 2.05 μm upon 120 min incubation). The respective ‘mean diameter’ of the DMCA-incubated WBCs exhibits a statistically significant increase in respect to that of the DMCA-free ones (16.13 ± 2.73 μm for C_DMCA_ = 0.1 mg/ml and 16.52 ± 2.68 μm for C_DMCA_ = 1 mg/ml, both upon 120 min incubation). Finally, the ‘mean diameter’ of the DMCA-incubated WBCs exhibits a borderline statistically significant increase.

Both DMCA concentrations used in our study exceed the safety levels recommended for the intravenous injection of iron agents in treatment of iron-deficiency anemia (i.e. C_Fe(III)_ = 0.016 mg/ml). The OM data refer to many hundreds of WBCs (approximately 720 for each donor) and indicate that, for the conditions studied here, the presence of the ^68^Ga-DPD-Fe_3_O_4_ DMCA promotes WBC activation by size and shape change from a spherical to a polarized and/or ruffled form.

**Figure 4:**
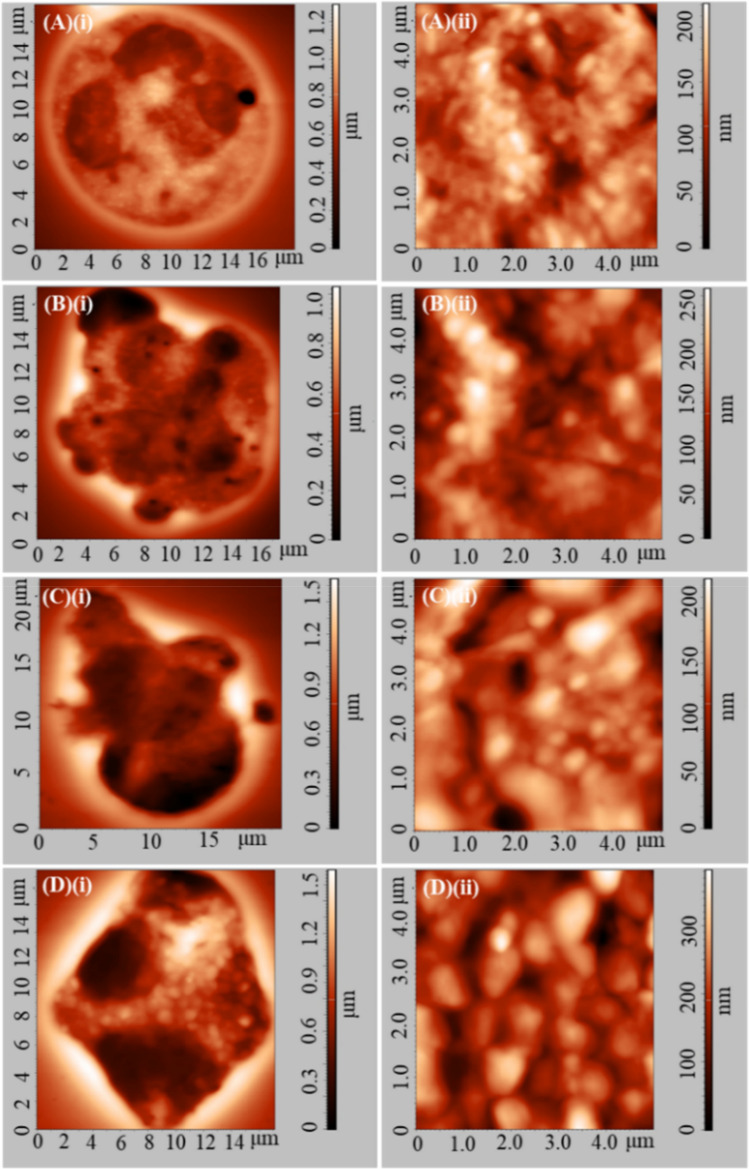
Representative two-dimensional atomic force microscopy (AFM) images of WBCs in resting and activated state. (**A**)(**i**)–(**D**)(**i**) Two-dimensional topography of WBCs referring to (**A**)(**i**) DMCA-free prior to incubation (0 min), (**B**)(**i**) DMCA-free after incubation (120 min), (**C**)(**i**) DMCA-incubated at C_DMCA_ = 0.1 mg/ml (120 min) and (**D**)(**i**) DMCA-incubated at C_DMCA_ = 1 mg/ml (120 min) samples. Panel (**A**)(**i**) shows a WBC in resting state, while panels (**B**)(**i**)–(**D**)(**i**) show WBCs in activated state. Panels (**A**)(**ii**)–(**D**)(**ii**) present surface granules of WBCs shown in (**A**)(**i**)–(**D**)(**i**) respectively, at a scanned area of 5 × 5 μm^2^. A typical comparison of (**A**)(**ii**)–(**D**)(**ii**) images reveals that the size of the WBC granules is increased in the activated state.

Figure 4A–D show representative two-dimensional topography AFM images of WBCs in (A)(i) resting state (coming from a DMCA-free prior to incubation (0 min) sample) and in (B)(i)–D(i) activated state. Panels (B)(i), (C)(i) and D(i) refer to a DMCA-free sample after incubation (120 min), a DMCA-incubated sample for C_DMCA_ = 0.1 mg/ml (120 min) and a DMCA-incubated sample for C_DMCA_ = 1 mg/ml (120 min), respectively. In all cases the morphological characteristics are recorded in detail both qualitatively and quantitatively at the nanoscopic level by means of AFM. As indicated in panel (A)(i) a resting WBC exhibits a completely spherical shape, whereas activated WBCs (panels (B)(i)–D(i)) undergo a noticeable morphological transformation, showing a polarized and/or ruffled shape. The panels (A)(ii)–(D)(ii) present surface granules of the respective WBCs at a scanned area of 5 × 5 μm^2^. A direct comparison of these panels shows that the granules of the WBCs increase in the activated state. These AFM data exhibit the typical behavior observed in all five donors.

**Figure 5:**
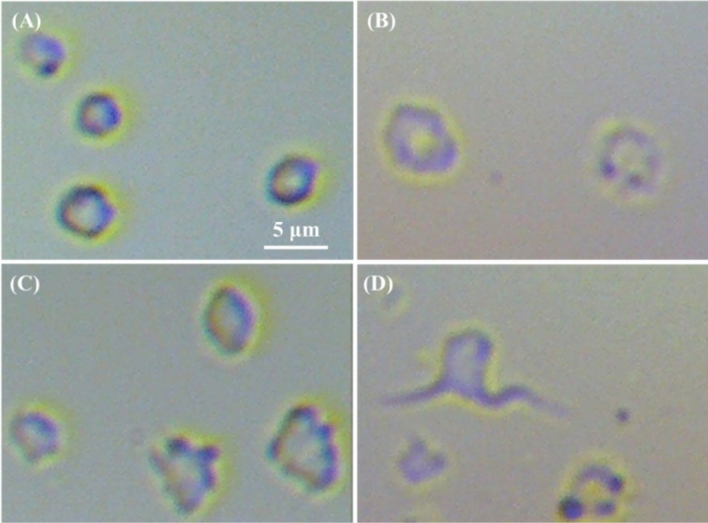
Representative optical microscopy (OM) images of PLTs incubated with and without the DMCA. (**A**)–(**D**) OM images of PLTs films referring to (**A**) DMCA-free prior to incubation (0 min), (**B**) DMCA-free after incubation (120 min), (**C**) DMCA-incubated at C_DMCA_ = 0.1 mg/ml (120 min) and (**D**) DMCA-incubated at C_DMCA_ = 1 mg/ml (120 min) (objective lens × 80, camera × 13 in all cases). A typical comparison of (**A**)–(**D**) images reveals that the shape of the PLTs progressively change in the presence of the DMCA from a nearly spherical form to an irregular one with the degree of activation (polarized and/or ruffled shape with the occasional appearance of pseudopods).

Figure 5A–D show representative OM images of PLT films coming from donor B for DMCA-free prior to (panel (A)) and after 120 min incubation (panel (B)) and DMCA-incubated samples for 120 min at room temperature in tolerable and extreme concentrations, C_DMCA_ = 0.1 mg/ml (panel (C)) and C_DMCA_ = 1 mg/ml (panel (D)), respectively. The direct comparison of these images shows that, at the microscopic level, the shape of PLTs progressively changes by the presence of the DMCA from a nearly spherical shape (panel (A)) to an irregular one with the degree of activation. Indeed, after 120 min incubation without (panel (B)) and with the DMCA (panels (C) and (D)) PLTs present a polarized and/or ruffled shape with the occasional appearance of pseudopods. The same behavior was recorded in all five donors studied here.

**Figure 6:**
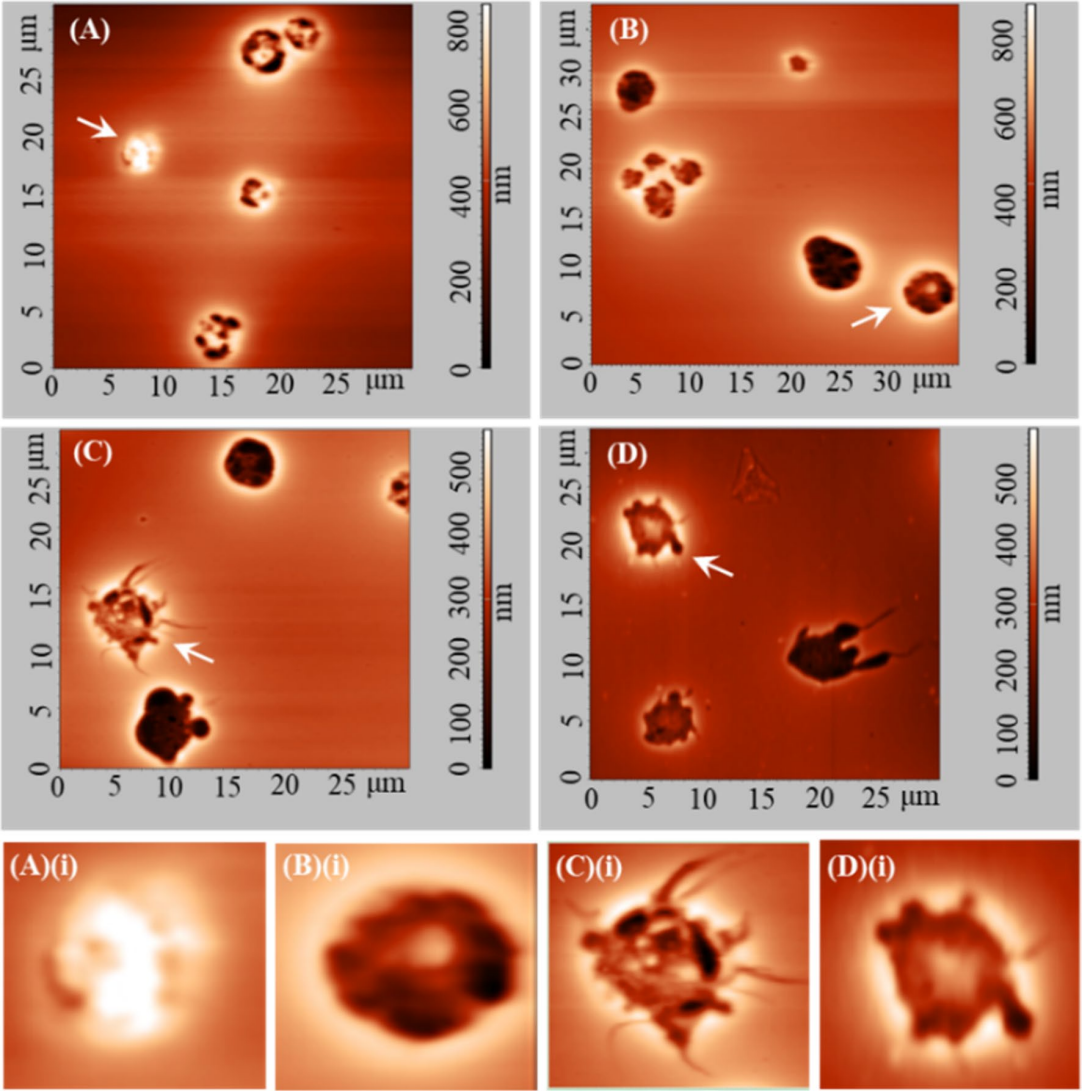
Representative two-dimensional atomic force microscopy (AFM) images of PLTs incubated with and without the DMCA. (**A**–**D**) Two-dimensional AFM images of PLTs referring to (**A**) DMCA-free prior to incubation (0 min), (**B**) DMCA-free after incubation (120 min), (**C**) DMCA-incubated at C_DMCA_ = 0.1 mg/ml(120 min) and (**D**) DMCA-incubated at C_DMCA_ = 1 mg/ml (120 min). (**A**)(**i**)–(**D**)(**i**) Bottom figures show magnification of specific PLTs indicated by the arrows in the respective top images, in (**A**)(**i**) resting (spherical shape) and in (**B**)(**i**)–(**D**)(**i**) activated state. A typical comparison of (**A**)–(**D**) and (**A**)(**i**)–(**D**)(**i**) images reveals that the shape of the PLTs changes in the presence of DMCA from a nearly spherical form to an irregular one with the degree of activation (polarized and/or ruffled shape with the occasional appearance of pseudopods).

Figure 6A–D show two-dimensional AFM images of PLTs referring to DMCA-free prior to incubation (0 min) (panel (A)), DMCA-free after incubation (120 min) (panel (B)), DMCA-incubated at C_DMCA_ = 0.1 mg/ml(120 min) (panel (C)) and DMCA-incubated at C_DMCA_ = 1 mg/ml (120 min) samples (panel (D)). Interestingly, the AFM images provide detailed and accurate information of the PLT shape morphology, which is recorded both qualitatively and quantitatively at the nanoscopic level. Panels (A)(i)–(D)(i) show in detail the change in shape morphology of specific PLTs, indicated by the arrows in the respective top images (panels (A)–(D)) upon activation. In particular, a PLT in resting state (panel (A)(i)) presents a nearly spherical shape, whereas it changes by the presence of DMCA to a polarized and/or ruffled one with the occasional appearance of pseudopods (panels (B)(i)–(D)(i)) upon activation. These data show the mean behavior that resulted from all five donors.

The AFM data refer to many hundreds of PLTs (approximately 320 for each donor) and indicate that the presence of the ^68^Ga-DPD-Fe_3_O_4_ DMCA promotes the PLTs’ activation by a shape change from a spherical to a polarized and/or ruffled form with the occasional appearance of pseudopods.

**Figure 7:**
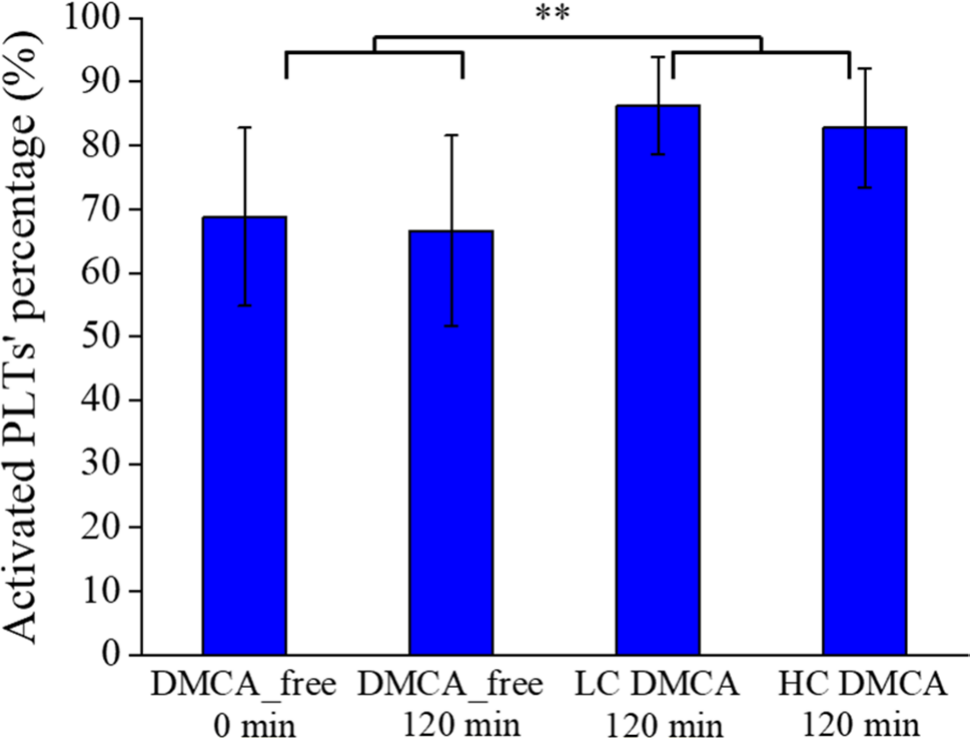
Percentage [%] of activated PLTs’ observed in samples incubated with and without the DMCA. Mean value of the activated PLTs’ percentage [%] observed in DMCA-free (prior to (0 min) and after (120 min) incubation) and DMCA-incubated (C_DMCA_ = 0.1 mg/ml; low concentration—LC and C_DMCA_ = 1 mg/ml; high concentration—HC) samples. In all panels the results are shown as the overall MV ± SD of the data coming from all five donors.

Figure 7 quantitatively summarizes the AFM results referring to the shape change of the activated PLTs observed in the DMCA-free and DMCA-incubated samples with different DMCA concentrations (C_DMCA_ = 0.1 mg/ml; low concentration—LC and C_DMCA_ = 1 mg/ml; high concentration—HC). Specifically, we observed that the PLTs are not activated during 120 min incubation without DMCA (68.8 ± 13.9% prior to versus 66.6 ± 14.9% after), whereas 120 min incubation with DMCA motivates activation (86.2 ± 7.7% for C_DMCA_ = 0.1 mg/ml and 82.8 ± 9.4% for C_DMCA_ = 1 mg/ml), without being concentration dependent.

Thus, from the obtained OM and AFM data, we can assume that both at the microscopic and at the nanoscopic level the activation of PLTs is promoted by the presence of the DMCA, as evidenced by a shape change from a nearly spherical to an irregular form (polarized and/or ruffled shape with the occasional appearance of pseudopods). We stress that despite the observed activation, no PLTs clustering was observed in any of the samples.

**Figure 8:**
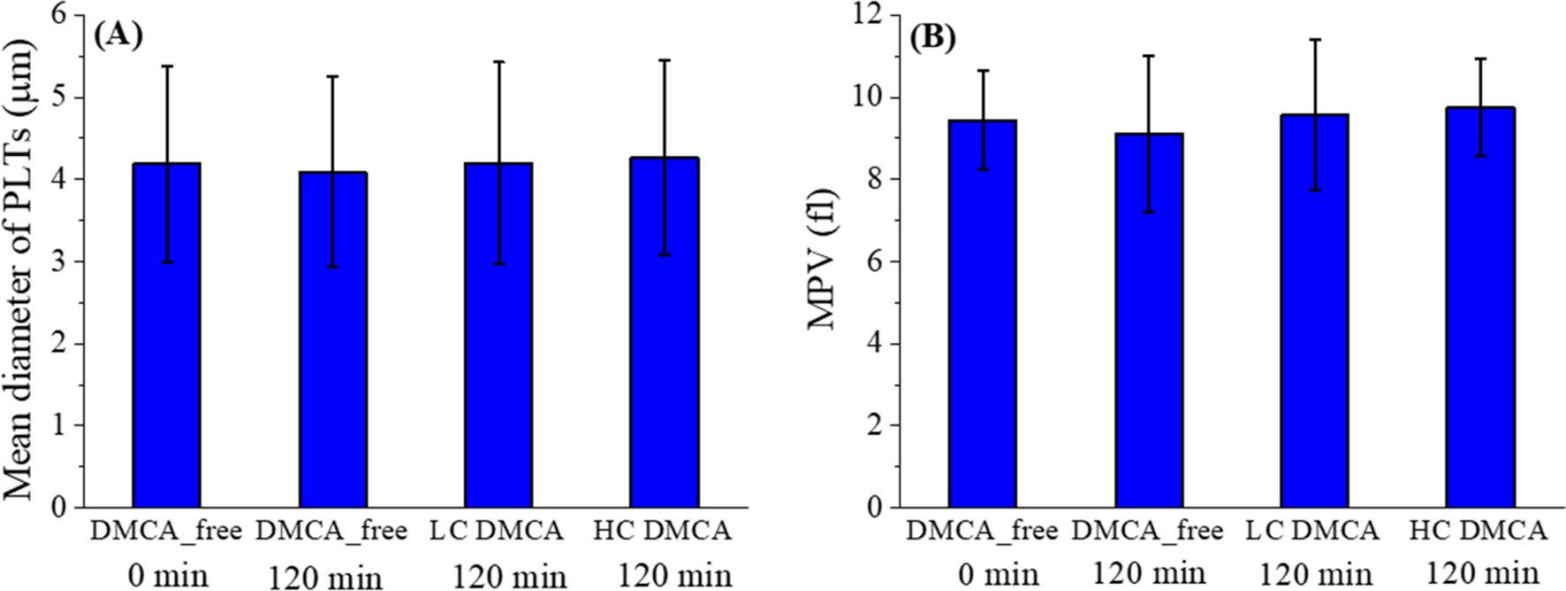
Quantitative data of PLTs’ size characteristics measured in all five donors by AFM. Mean value of the PLTs’ size characteristics, namely (**A**) diameter and (**B**) mean platelet volume (MPV), measured in DMCA-free (prior to (0 min) and after (120 min) incubation) and DMCA-incubated (C_DMCA_ = 0.1 mg/ml; low concentration—LC and C_DMCA_ = 1 mg/ml; high concentration—HC) samples. In both panels the results are shown as the overall MV ± SD of the data coming from all five donors.

Figure 8 quantitatively summarizes the AFM results of the size characteristics, mean diameter (panel A) and mean platelet volume (MPV) (panel B), of the PLTs measured in the DMCA-free and DMCA-incubated samples with different DMCA concentrations (C_DMCA_ = 0.1 mg/ml; low concentration—LC and C_DMCA_ = 1 mg/ml; high concentration—HC). First, we clarify that in an activated PLT the parameter ‘mean diameter’ refers to the mean value of the two diameters as measured along the basic axis of elongation and the normal direction. Thus, in non-activated state, the ‘mean diameter’ coincides to the typical diameter. On the other hand, the mean platelet volume (MPV) of a PLT was calculated based on the AFM data (for details, see the “Definition of activated cells” in “Methods”).

Referring to the diameter (panel (A)) of PLTs measured in all samples, we observed that it is practically identical (4.19 ± 1.19 for DMCA_free (0 min), 4.09 ± 1.16 for DMCA_free (120 min), 4.20 ± 1.23 for C_DMCA_ = 0.1 mg/ml and 4.27 ± 1.19 for C_DMCA_ = 1 mg/ml). The same result stands for the MPV (9.44 ± 1.20 for DMCA_free (0 min), 9.12 ± 1.91 for DMCA_free (120 min), 9.57 ± 1.84 for C_DMCA_ = 0.1 mg/ml and 9.76 ± 1.18 for C_DMCA_ = 1 mg/ml). Thus, we can assume that the PLTs’ overall size characteristics (diameter and volume) do not change upon activation.

Finally, we would like to comment on the relatively high initial value of PLTs’ activation percentage (67%) observed in the DMCA-free samples prior to incubation (0 min) as evidenced in Fig. 7. This fact can be ascribed to the non-ideal conditions that hold during every in vitro process. First, PLTs are stored at room temperature (20–25 °C) that differs substantially from the ideal body temperature (36–37 °C). Second, when left on the workbench the PLTs are at rest that is they are not subjected to the ideal flow conditions that experience in the cardiovascular system. Third, other causes such as the strength of centrifugation during the stage of PLTs isolation may play an important role. The non ideal temperature storage^32–34^, absence of agitation (as well as type and strength of agitation)^35–37^ and strength of centrifugation^38,39^ may all influence the status of PLTs, ultimately leading to a relatively high baseline activation. However, the aim of our study is to observe if any pre-existing activation of PLTs is promoted by the presence of the DMCA. Indeed, this is evidenced by the data of Fig. 7.

To obtain further information for the status of many thousands of WBCs and PLTs, we recruited an HA, which is employed in every day clinical practice. The Table 1 presents this information for DMCA-free (prior to and after incubation) and DMCA-incubated (C_DMCA_ = 0.1 mg/ml; low concentration—LC and C_DMCA_ = 1 mg/ml; high concentration—HC) samples. The complete blood count showed that all standard WBCs and PLTs indices remained in the physiological range, even in the DMCA-incubated sample with the high concentration of C_DMCA_ = 1 mg/ml. Thus, we can assume that despite the observed morphological activation by means of OM and AFM, the presence of ^68^Ga-Fe_3_O_4_-DPD DMCA had no intense impact on WBCs and PLTs.

**Table 1:** Main WBCs’ and PLTs’ indices obtained by a clinical hematology analyzer (HA).

	DMCA-free (t = 0 min)	DMCA-free (t = 120 min)	LC DMCA (t = 120 min)	HC DMCA (t = 120 min)
WBC 4.0–10.0 (K/μL)	6.0 ± 1.0	4.8 ± 1.6	5.5 ± 1.5	5.9 ± 1.1
PLT 150–400 (K/μL)	266 ± 50	268 ± 35	240 ± 92	254 ± 68
PCT 0.16–0.35 (%)	0.26 ± 0.06	0.27 ± 0.09	0.23 ± 0.11	0.25 ± 0.07
MPV 7.2–11.0 (fl)	9.7 ± 2.0	10.0 ± 2.2	9.8 ± 2.5	10.2 ± 2.4

HA data referring to standard WBCs’ and PLTs’ indices, namely concentration of white blood cells (WBC), concentration of platelets (PLT), plateletcrit (PCT) and mean platelet volume (MPV), of the DMCA-free (prior to and after incubation) and DMCA-incubated (C_DMCA_ = 0.1 mg/ml; low concentration—LC and C_DMCA_ = 1 mg/ml; high concentration—HC) samples for 120 min at room temperature. The results are expressed as the mean value ± standard deviation (MV ± SD) of five healthy donors.

Combining all the OM, AFM and HA data presented above for both WBCs and PLTs, we can conclude that although the presence of the DMCA promoted their activation, neither degradation nor clustering were observed, hence making this DMCA a potential candidate for diagnostic imaging. However, these results are exclusively in vitro. Clearly, in vivo data is highly necessitated, so that safer conclusions can be drawn. To this effect, we studied nine Swiss and nine SCID mice on a comparative basis under administration of a standard DMCA dose (see subsection “In vivo biodistribution study on normal and immunodeficient mice” of section “Methods”).

**Figure 9:**
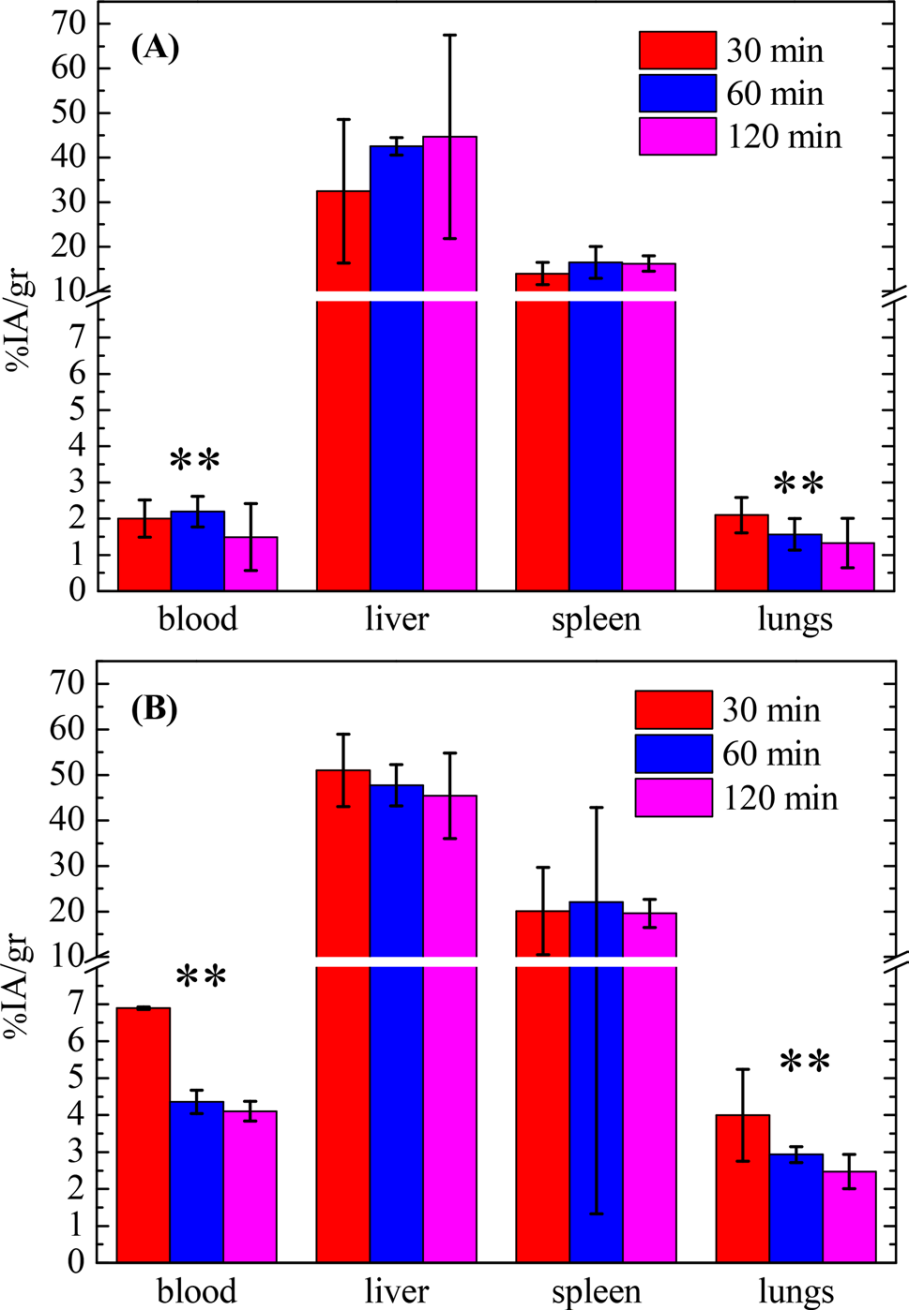
Accumulation of the DMCA in the blood and the MPS organs of normal Swiss and immunodeficient SCID mice. The accumulation of the DMCA in the blood and in the organs of the mononuclear phagocyte system (MPS) as percentage injected activity per gram of tissue (%IA/g) at 30, 60 and 120 min post injection in (a) Swiss and in (b) SCID mice. For each time point three mice were studied, thus the results are expressed as the MV ± SD.

Figure 9 presents quantitative data of the accumulation of the DMCA in the blood and in the main organs of the mononuclear phagocyte system, namely liver, spleen and lungs, as percentage of the injected activity per gram of tissue (%IA/g) at 30, 60 and 120 min post injection in Swiss (panel (A)) and in SCID (panel (B)) mice. Specifically, the blood retention of the DMCA in Swiss mice exhibits an overall statistically significant difference when compared to the one in SCID mice (2.00 ± 0.51, 2.19 ± 0.42 and 1.49 ± 0.92 versus 6.89 ± 0.04, 4.36 ± 0.31 and 4.10 ± 0.27, at 30, 60 and 120 min post injection, respectively). On the contrary, the %IA/gof the DMCA in the liver in both Swiss and SCID mice is practically identical (32.42 ± 16.12, 42.52 ± 1.95 and 44.66 ± 22.86 versus 51.00 ± 7.93, 47.74 ± 4.56 and 45.42 ± 9.38, at 30, 60 and 120 min post injection). The same holds for the %IA/g of the DMCA in the spleen in both Swiss and SCID mice (13.98 ± 2.49, 16.48 ± 3.58 and 16.21 ± 1.74 versus 20.04 ± 9.62, 22.08 ± 20.75 and 19.57 ± 3.10). Finally, referring to the %IA/g of the DMCA in the lungs, we see that the respective values in Swiss mice exhibits an overall statistically significant difference when compared to the one in SCID mice (2.10 ± 0.49, 1.57 ± 0.43 and 1.32 ± 0.69 versus 3.99 ± 1.24, 2.93 ± 0.21 and 2.47 ± 0.47).

The above data evidenced high and practically equal uptake of the DMCA in the liver and spleen in both animal models. To explain these data we recall that the immune system is divided into the innate and the adaptive. The former is the first defense mechanism exhibiting a non-specific response against a pathogen, while the latter is the second defense mechanism and is characterized by antigen specificity and immunologic memory. In addition, organs rich in phagocytic cells, such as liver and spleen, constitute an important part of the innate immune system, while organs rich in T and B lymphocytes, such as spleen and lungs, are major components of the adaptive^40^. Also, we recall that the nanoparticles are quickly opsonized upon their administration into the bloodstream, which is the main reason of their recognition and elimination by the phagocytic cells of innate system.

In our case, normal Swiss mice show physiological function of the entire immune system both innate and adaptive, compared to the ones bearing the SCID mutation, which are devoid of T and B cells, hence the adaptive system. Accordingly, the comparative results obtained in our study demonstrate that the particular DMCA encounters the activation of the innate immune system through the action of phagocytic cells, at least in the mice models studied here. This is an encouraging first indication that the ^68^Ga-DPD-Fe_3_O_4_ DMCA studied here may be a safe candidate for further in vivo applications in humans.

**Figure 10:**
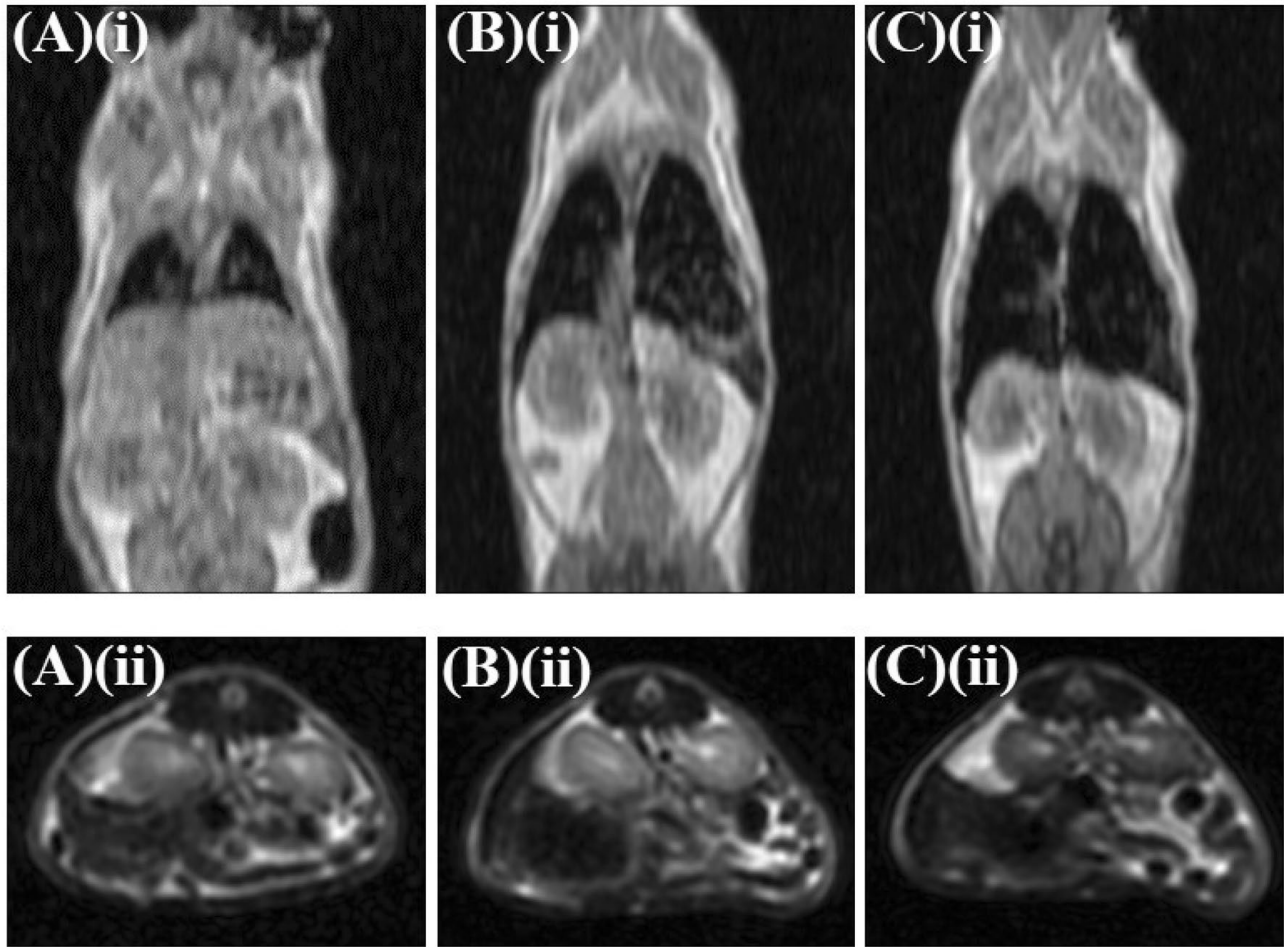
Representative T_1_-weighted coronal and T_2_-weighted axial MRI data of normal Swiss mice. (**A**)(**i**)–(**C**)(**i**) T_1_-weighted coronal MRI data of n = 3 normal Swiss mice injected with (**A**)(**i**)–(**B**)(**i**) ^68^Ga-DPD-Fe_3_O_4_ DMCA at different concentrations: (**A**)(**i**) C_DMCA_ = 0.01 mg/ml and (**B**)(**i**) C_DMCA_ = 0.1 mg/ml, respectively and with (**C**)(**i**) non-radiolabeled DPD-Fe_3_O_4_ CA at a concentration of C_CA_ = 0.3 mg/ml. (**A**)(**ii**)–(**C**)(**ii**) T_2_-weighted axial MRI data of n = 3 normal Swiss mice injected with (**A**)(**ii**)–(**B**)(**ii**) ^68^Ga-DPD-Fe_3_O_4_ DMCA at different concentrations: (**A**)(**ii**) C_DMCA_ = 0.01 mg/ml and (**B**)(**ii**) C_DMCA_ = 0.1 mg/ml, respectively and with (**C**)(**ii**) non-radiolabeled DPD-Fe_3_O_4_ CA at a concentration of C_CA_ = 0.3 mg/ml. All MRI images were acquired 6 h p.i.

In vivo MRI experiments were performed on three normal Swiss mice to evaluate the efficacy of the DMCA, ^68^Ga-DPD-Fe_3_O_4_, on a comparative basis to that of the parent non-radiolabeled CA, DPD-Fe_3_O_4_. Figure 10 illustrates representative T_1_-weighted coronal (panels (A)(i)–(C)(i)) and T_2_-weighted axial (panels (A)(ii)–(C)(ii)) MRI data of n = 3 normal Swiss mice focused on the area of interest (namely, liver and spleen). The mice were injected with the ^68^Ga-DPD-Fe_3_O_4_ DMCA at two different concentrations, C_DMCA_ = 0.01 mg/ml (panels (A)(i) and (A)(ii)) and C_DMCA_ = 0.1 mg/ml (panels (B)(i) and (B)(ii)), respectively, and with the parent, non-radiolabeled DPD-Fe_3_O_4_ CA at a concentration of C_CA_ = 0.3 mg/ml (panels (C)(i) and (C)(ii)). All MRI data were acquired 6 h p.i. A comparison of the MRI data (A)(i)/(A)(ii) with the respective ones (B)(i)/(B)(ii) reveals that the contrast effect of the DMCA is clearly enhanced (loss of signal) upon increase of its dose. This is due to the accumulation of the DMCA in the liver and spleen and indicates that the contrast effect of the DMCA is concentration-dependent, as expected. This finding is in nice agreement to the respective in vivo biodistribution data presented above in Fig. 9A. Also, a comparison of the MRI data (B)(i)/(B)(ii) with the respective ones (C)(i)/(C)(ii) reveals that the contrast effect of the DMCA, ^68^Ga-DPD-Fe_3_O_4_, at the tolerable concentration C_DMCA_ = 0.1 mg/ml, is comparable to that of the parent, non-radiolabeled CA, DPD-Fe_3_O_4_, at a three-times higher concentration C_CA_ = 0.3 mg/ml. This result is significant since it proves that the magnetic properties of the parent CA, DPD-Fe_3_O_4_, are not suppressed by the radiolabeling process. Accordingly, the resulted DMCA is clearly efficient for both PET (Ref.^27^) and MRI (this work).

## Conclusion

In the current study, we examined the biocompatibility of a new DMCA showing in vitro biopsy results on immune and coagulation cells of human blood and comparative in vivo biodistribution data on normal and immunodeficient mice.

Referring to the in vitro experiments, donated WBCs and PLTs from five healthy individuals were incubated at room temperature for 120 min with two different concentrations of the DMCA (tolerable/low concentration, C_DMCA_ = 0.1 mg/ml and extreme/high concentration, C_DMCA_ = 1 mg/ml). The evaluation of the shape/size characteristics of WBCs and PLTs was performed by means of OM and AFM, in order to obtain information from both the microscopic and the nanoscopic level. The results showed that the shape and size of WBCs, as well as the shape of PLTs change at the microscopic/nanoscopic level in the presence of the DMCA. Specifically, at the microscopic level, we observed that WBCs are noticeably activated during 120 min incubation without DMCA (27.6 ± 13.8% prior to versus 57.6 ± 15.5% after), whereas 120 min incubation with DMCA enhances activation (79.2 ± 3.3% for C_DMCA_ = 0.1 mg/ml and 78.8 ± 5.4% for C_DMCA_ = 1 mg/ml), as proved by a shape change from a completely spherical to a polarized and/or ruffled form. Furthermore, the ‘mean diameter’ of WBCs changes with their activation due to the presence of the DMCA (14.38 ± 1.84 μm (prior to) and 14.53 ± 2.05 μm (after incubation), versus 16.13 ± 2.73 μm for C_DMCA_ = 0.1 mg/ml and 16.52 ± 2.68 μm for C_DMCA_ = 1 mg/ml).

At the nanoscopic level, we observed that the size of the WBCs granules is increased in the activation state. Concerning the PLTs, at the microscopic level, we observed that they are not activated during 120 min incubation without DMCA (68.8 ± 13.9% prior to versus 66.6 ± 14.9% after), whereas 120 min incubation with DMCA motivates activation (86.2 ± 7.7% for C_DMCA_ = 0.1 mg/ml and 82.8 ± 9.4% for C_DMCA_ = 1 mg/ml), as proved by a shape change from a completely spherical to a polarized and/or ruffled form with the occasional appearance of pseudopods. However, despite the activation of both the WBCs and PLTs, neither degradation nor clustering was observed. These findings were also enhanced by the HA data, which showed that all standard WBC and PLT indices remained in the physiological range. The in vitro results indicate adequate biocompatibility of the particular DMCA with human WBCs and PLTs.

Referring to the in vivo experiments, comparative biodistribution studies were performed between normal Swiss and SCID mice to evaluate the accumulation of the DMCA at the main organs of the mononuclear phagocyte system. The overall results indicated high accumulation of the DMCA in organs rich in phagocytic cells, namely liver and spleen, without any statistical difference between the two mice groups. In vivo MRI imaging studies in normal Swiss mice were performed to evaluate the efficacy of the DMCA. The results indicated that the contrast effect produced by the particular DMCA was concentration-dependent, as expected. More importantly, the contrast efficacy of the DMCA, ^68^Ga-DPD-Fe_3_O_4_, was clearly comparable to that of the parent, non-radiolabeled CA, DPD-Fe_3_O_4_.

The overall in vitro and in vivo data presented here prove that the DMCA studied here is a promising candidate for future applications.

## Methods

### Preparation of ^68^Ga-DPD-Fe_3_O_4_ DMCA

The synthesis and radiolabeling of ^68^Ga-DPD-Fe_3_O_4_ DMCA have been reported elsewhere^27^. For obvious safety reasons all experiments with ^68^Ga-DPD-Fe_3_O_4_ DMCA were conducted after the decay of Gallium-68 to the nonradioactive isotope Zinc-68.

### Healthy donors and blood collection

All the donors participated in this study provided their written, informed consent. The donors underwent blood sampling following the approval of the Ethics Committee of the NCSR “Demokritos”. All experiments were carried out in accordance with relevant guidelines and regulations. In brief, five donors coming from the authors and colleagues participated this study after a screening complete blood count was conducted (sex: 2 men and 3 women and age: 20–60 years old)^31^. One donor was hypertensive and followed relevant medication by the time of the study (Preterax, 5 mg/1.25 mg). The other four donors had no indication/confirmation of any current/chronic disease. None of the five donors had any hematological disease. Accordingly, all five donors participating this study are considered to be healthy.

According to our study, 15 ml of whole blood was drawn by venipuncture from each donor, which was equally deposited in five anticoagulant-containing ethylenediaminetetraacetic acid (EDTA) Vacutainer K3 test tubes (BD, Franklin Lakes, NJ, USA).Since our experimental protocol requires all blood samples to be fresh, the whole blood was used directly after its collection, without being stored^28–31^.

### Isolation of the buffy coat, incubation process and preparation of WBC and PLT films

For the effective study of the WBCs and PLTs with the OM and AFM techniques, single layered films of appropriate characteristics should be prepared^28–30^. To this end, an amount of 15 ml of whole blood was drawn by venipuncture from each donor and was equally distributed in five EDTA test tubes that were left on the work bench at room temperature for 1 h so that blood cells were mildly fractionated under the gravity force. As a result, a precipitate of RBCs was formed at the bottom of the tube, while the supernatant plasma was rich primarily in PLTs and secondarily in WBCs (platelets rich plasma) with a rich reservoir primarily of WBCs and secondarily of PLTs agglomerated at the interface of these two fractions (buffy coat). Then, the buffy coat was collected via careful aspiration.

After the isolation of the buffy coat, ten parts of each sample was incubated with one part of ^68^Ga-DPD-Fe_3_O_4_ DMCA at low (C_DMCA_ = 0.1 mg/ml) and high (C_DMCA_ = 1 mg/ml) concentrations^31^. The incubation process was performed under mild conditions (20 rounds per minute) at room temperature (T = 25 °C) for 120 min. We note that this process exerts minimum mechanical stress on WBCs and PLTs, thus preserving their morphological characteristics at the maximum degree possible. DMCA-free WBCs and PLTs prior to and after incubation (under the same conditions with the DMCA-loaded samples) served as control samples.

Accordingly, four distinct samples’ categories were prepared and studied for each one of the donors: DMCA-free WBCs and PLTs prior to incubation, DMCA-free WBCs and PLTs after incubation, DMCA-incubated WBCs and PLTs at tolerable/low concentration (C_DMCA_ = 0.1 mg/ml) and DMCA-incubated WBCs and PLTs at extreme/high concentration (C_DMCA_ = 1 mg/ml). For each one of these categories three copies of dry films were prepared onto standard glass slides as described elsewhere^28–30^ for examination with OM and AFM.

### Optical microscopy

OM images of WBC and PLT films were acquired by using a standard light microscope (LEICA DMRXP, Leica, Wetzlar, Germany) in transmission mode operation with an overall magnification up to 1000 ×. From the obtained OM images microscopic-level information was extracted on the overall shape morphology of the WBCs and PLTs. For each donor, approximately n = 180 WBCs and n = 150 PLTs were observed per sample. Given that four different categories of samples were studied in each case this makes a total population of n = 720 WBCs and n = 600 PLTs for each donor.

### Atomic force microscopy

AFM images of WBC and PLT films were recorded using a scanning probe microscope (Solver PRO, NT-MDT Co, Moscow, Russia) performing in non-contact scanning mode. Detailed information on the AFM instrumentation can be found in^31^. The optimum imaging results were acquired by setting the following scanning parameters: line frequency = 1.5–4 Hz, area = 0.5 × 0.5–150 × 150 μm^2^ and lines per image = 256–512^31^. For each donor, approximately n = 15 WBCs and n = 80 PLTs were scanned per sample and detailed information on morphological characteristics, mainly shape and size, were obtained at the nanoscopic level. Given that four different categories of samples were studied in each case this makes a total population of n = 60 WBCs and n = 320 PLTs for each donor.

### Definition of activated cells

**Figure 11:**
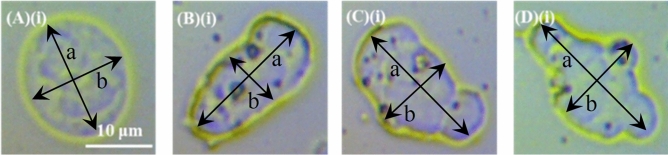
Quantitative criterion of polarization for resting and activated WBCs. (**A**)(**i**)–(**D**)(**i**) OM images of WBCs in (**A**)(**i**) resting (spherical shape) and in (**B**)(**i**)–(**D**)(**i**) activated state, where the long (a) and short (b) axes of the cells are indicated by the arrows for each case. The quantitative criterion of polarization was based on the dimensionless ratio (a − b)/a and was found to be 0.15 for (**A**)(**i**), 0.52 for (**B**)(**i**), 0.44 for (**C**)(**i**) and 0.35 for (**D**)(**i**).

The activated WBCs were counted according to their shape change, which was evident by the OM. The activated WBCs were defined by using the quantitative criterion of polarization (else flattening/ellipticity) based on the dimensionless ratio (a − b)/a, where a and b are the long and short axes of the cell. In Fig. 11, the procedure for the cells already presented in Fig. 1(A)(i)–(D)(i), is demonstrated. The results on polarization are 0.15, 0.52, 0.44 and 0.35 for each case. Taking into account standard sources of error, the threshold criterion we used for distinguishing non-activated and activated cells is the value 0.2; for polarization below 0.2 the cell was considered as non-activated, while when the polarization exceeded 0.2 the cell was considered activated.

**Figure 12:**
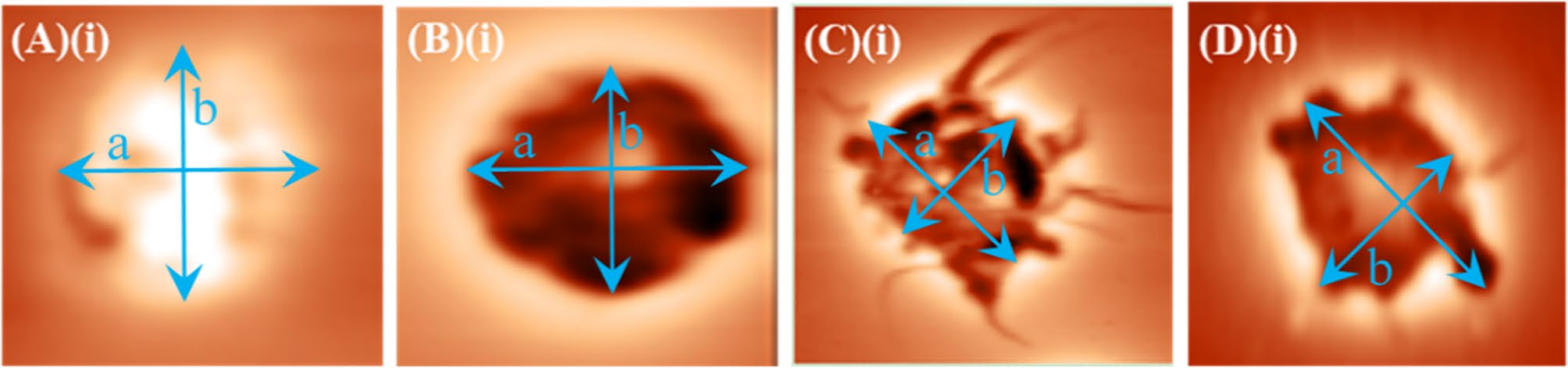
Quantitative criterion of polarization for resting and activated PLTs. (**A**)(**i**)–(**D**)(**i**) OM images of PLTs in (**A**)(**i**) resting (spherical shape) and in (**B**)(**i**)–(**D**)(**i**) activated state, where the long (a) and short (b) axes of the cells are indicated by the arrows for each case. The quantitative criterion of polarization was based on the dimensionless ratio (a − b)/a and was found to be 0.03 for (**A**)(**i**), 0.19 for (**B**)(**i**), 0.37 for (**C**)(**i**) and 0.46 for (**D**)(**i**).

The activated PLTs were counted according to their shape change, which was evident by the AFM. The activated PLTs were defined by using the same quantitative criterion of polarization as in the case of WBCs, namely the ratio (a − b)/a. In Fig. 12, the procedure for the cells already presented in Fig. 6(A)(i)–(D)(i), is demonstrated. The results on polarization are 0.03, 0.19, 0.37 and 0.46, for each case. Taking into account standard sources of error, the threshold criterion we used for distinguishing non-activated and activated PLTs is the value 0.13; for polarization below 0.13 the cell was considered as non-activated, while when the polarization exceeded 0.13 the cell was considered activated. On the other hand, the mean platelet volume (MPV) of a PLT was calculated based on the quantitative data of AFM.

We have minimized the possibly subjective nature of the quantitative analysis of the OM and AFM results due to the following two reasons: (i) the exact quantitative criterion on the polarization mentioned above that was used in common for all sample series guarantees an objective comparative basis and (ii) the former quantitative analysis was conducted independently by the three individuals and the results were practically identical.

### Clinical hematology analyzer

Reference samples of WBCs and PLTs incubated with and without the DMCA underwent complete blood count by means of a standard clinical hematology analyzer (ADVIA 560 Hematology System, Siemens Healthcare GmbH, Erlangen, Germany). Given that the population of WBCs and PLTs examined by means of OM and AFM was inevitably limited (800–1000 cells), the utilization of an HA was crucial for confirmation reasons. Accordingly, the HA provided information concerning all standard WBC and PLT indices that stems from the examination of many thousands of WBCs and PLTs^31^.

To test the reliability of the HA and to reject any possible interference with the DMCA nanoparticles, control tests were performed on representative dummy samples. Specifically, blood plasma, DMCA-loaded blood plasma and DMCA-loaded distilled H_2_O were examined. We safely concluded that the size of the DMCA nanoparticles (< 200 nm)^27^ is well below the detection limit of the HA, thus their presence does not influence the recorded data.

### In vivo biodistribution study on normal and immunodeficient mice

Animal experiments were carried out according to European and national regulations. These studies have been further approved by the Ethics Committee of the NCSR “Demokritos” and animal care and procedures followed are in accordance with institutional guidelines and licenses issued by the Department of Agriculture and Veterinary Policies of the Prefecture of Attiki (Registration numbers: EL 25 BIO 022 and EL 25 BIO 021). Normal Swiss and immunodeficient SCID mice were obtained from the breeding facilities of the Institute of Biosciences and Applications, NCSR “Demokritos.” The study protocol was approved by the Department of Agriculture and Veterinary Policies of the Prefecture of Attiki (Protocol Number: 875110/11-11-2020).

The in vivo biodistribution of the DMCA was evaluated both in 9 Swiss mice (weight 23–27 g) and in 9 SCID mice (weight 17–19 g). To avoid venous thrombosis, prior to tail injection the sample of ^68^Ga-DPD-Fe_3_O_4_ DMCA was loaded onto a size exclusion PD-10 column, containing Sephadex G-25 resin and eluted with phosphate buffer saline (PBS), in order to eliminate aggregated ^68^Ga-DPD-Fe_3_O_4_ DMCA. For this reason, ten 0.5 ml fractions were collected, and the radioactivity of each fraction was measured using a dose calibrator (Capintec, Ramsey, NJ). The fractions containing the highest radioactivity were pooled and used for the study. Specifically, intravenous administration of 100 μl PBS suspension (11.11 μg/100 μl DMCA per mouse) of DMCA was performed via the tail vein. The animals were sacrificed at 30, 60, and 120 min post injection (3 Swiss and 3 SCID mice per time-point). Then, samples of blood and organs of interest that belong to the mononuclear phagocyte system, namely liver, spleen and lungs, were excised, weighed, and measured for radioactivity in a Gamma scintillation counter (Cobra II, Canberra, Packard, Downers Grove, Illinois). The remaining radioactivity in the tail, as well as background counts was subtracted, and the radioactivity decay was autocorrected by the counter. Then, the accumulation of the DMCA in each organ was expressed as the percentage injected activity per gram of tissue (%IA/g ± SD) and calculated compared to the activities of a standard dose of the injected solution.

### In vivo MRI imaging study on normal Swiss mice

The MRI imaging was conducted at the Alphavet Veterinary Diagnostic Imaging Center, Athens, Greece, using a 1.5 T MRI Unit. In brief, n = 3 normal Swiss mice (weight 31–33 g) were used for the MRI study, which were intravenously injected via the tail vein as follows: mouse (1) was injected with 100 μl (0.0221 mg) ^68^Ga-DPD-Fe_3_O_4_ DMCA, leading to a final body concentration of C_DMCA_ = 0.01 mg/ml, mouse (2) was injected with 100 μl (0.229 mg) ^68^Ga-DPD-Fe_3_O_4_ DMCA, leading to a final body concentration of C_DMCA_ = 0.1 mg/ml and mouse (3) was injected with 100 μl (0.8 mg) non-radiolabeled, DPD-Fe_3_O_4_ CA, leading to a final body concentration of C_CA_ = 0.3 mg/ml. The calculations of the final concentrations were based on the assumption that the blood content is approximately 7% of the total weight of each mouse. After 6 h p.i., the mice were anesthetized with an intraperitoneal injection of ketamine (75 mg/kg) and xylazine (5 mg/kg) and placed for imaging, where standard T_1_-weighted coronal and T_2_-weighted axial MRI data were acquired.

### Statistical analysis

Statistical analysis of the obtained results was performed by Pair Sample *t* test. Data are presented as mean value ± standard deviation (MV ± SD). Differences were considered not significant at P > 0.05 and statistically significant at P < 0.05 (**).
